# Draft Genome Sequence of Streptococcus agalactiae Strain AH2, Isolated from Hybrid Tilapia (Oreochromis niloticus × Oreochromis aureus)

**DOI:** 10.1128/mra.00747-22

**Published:** 2022-11-09

**Authors:** Ahmed H. Al-Harbi

**Affiliations:** a Life Science and Environment Research Institute, King Abdulaziz City for Science and Technology, Riyadh, Saudi Arabia; University of Rochester School of Medicine and Dentistry

## Abstract

This work describes the draft genome sequence of Streptococcus agalactiae strain AH2, which was isolated from moribund farmed hybrid tilapia (Oreochromis niloticus × Oreochromis aureus) in Saudi Arabia. The draft genome is composed of a single chromosome of 2,104,071 bp, with a GC content of 35.7%.

## ANNOUNCEMENT

Streptococcus agalactiae (group B streptococcus [GBS]) is a Gram-positive pathogenic bacterium that affects a number of terrestrial and aquatic animal species, including humans, camels, cattle, horses, dogs, monkeys, cats, mice, rabbits, frogs, fish, crocodiles, dolphins and other aquatic mammals (1–8). S. agalactiae strain AH2 was isolated in 2000 from moribund farmed hybrid tilapia (Oreochromis niloticus × Oreochromis aureus) in Saudi Arabia as described previously (9).

For DNA extraction, S. agalactiae AH2 was grown aerobically on tryptone soy agar (TSA) (Oxoid) for 24 h at 30°C, and a single colony was inoculated into 10 mL of tryptone soy broth (TSB) (Oxoid) and grown overnight at 30°C. The bacterial cells were harvested, the genomic DNA was extracted using the DNeasy blood and tissue kit (Qiagen), and the DNA quantity and quality were determined using a NanoDrop spectrophotometer (Thermo Fisher Scientific, USA) and 1% agarose gel electrophoresis, respectively.

Purified genomic DNA was subjected to draft genome shotgun sequencing using both Illumina and Pacific Biosciences (PacBio) platforms. For Illumina sequencing, genomic DNA was sheared using g-TUBEs (Covaris, Woburn, MA, USA) and purified using AMPure PB beads (Beckman Coulter), and a sequencing library with an average size of ~470 bp was prepared using the Nextera XT library preparation kit (Illumina, San Diego, CA, USA) and sequenced using 150-bp paired-end reads on the NovaSeq 6000 system. Reads were adapter trimmed using Trimmomatic v0.30 with a sliding window quality cutoff score of Q15 (10). Libraries were analyzed for fragment size using the 2100 Bioanalyzer (Agilent Technologies, Inc., Santa Clara, CA, USA) and quantified by quantitative PCR (qPCR) with a Kapa library quantification kit (Thermo Fisher Scientific). For PacBio sequencing, genomic DNA was sheared using g-TUBEs (Covaris), size selected to >10 kb using the BluePippin system, end repaired and ligated with universal hairpin adapters using the SMRTbell template preparation kit v1.0 (PacBio, Menlo Park, CA, USA) following the manufacturer’s instructions, and sequenced on the RSII platform (PacBio) using P6-C4 chemistry and 240-min movies.

Sequencing reads were filtered and *de novo* assembled using Flye v2.8 (11) and Mecat2 vJAN-2020 software run with default parameters. Multiple assembled results were merged with quickmerge and then Arrow with FALCON-Unzip. Clean reads were used for error correction with Pilon v1.22 (12) for the final genome assembly.

The quality and completeness of the assembled genome were assessed by Benchmarking Universal Single-Copy Orthologs (BUSCO) v4.1.4 (13) with the bacteria_odb10 data set (created 6 March 2020). This resulted in 98.4% complete genes, with 98.4% of the genome being single-copy genes, 0.0% duplicated genes, 1.6% fragmented genes, and 0.0% missing genes. The genomes were annotated by NCBI using the Prokaryotic Genome Annotation Pipeline (PGAP) v4.2 (14). Default parameters were used for all software unless otherwise specified.

The S. agalactiae AH2 draft genome was assembled into 3 contigs, ranging from 16,056 bp to 2,104,071 bp, which had a combined length of 2,146,970 bp, with an *N*_50_ value of 12,139 bp and a GC content of 36.3%. The chromosome was determined to be 2,104,071 bp long, with a GC content of 35.7%. The assembly statistics and genomic features are shown in Table 1.

**TABLE 1:** Genomic characteristics of Streptococcus agalactiae strain AH2

Characteristic	Value
Sequencing and assembly features	
No. of Illumina reads	7,983,500
No. of PacBio reads	372,294
Total no. of Illumina bases	1,174,604,400
Total no. of PacBio bases	4,160,012,139
Avg genome coverage (×)	1,693.35
Genome features	
Genome size (bp)	2,146,970
No. of contigs	3
GC content (%)	36.3
Total no. of genes	2,116
No. of coding sequences	2,012
No. of proteins	1,943
No. of pseudogenes	69
No. of rRNAs	21
No. of tRNAs	80
No. of noncoding RNAs	3

### Data availability.

The draft genome sequence of S. agalactiae strain AH2 has been deposited in DDBJ/ENA/GenBank under accession number JAKDEZ000000000, BioProject accession number PRJNA791657, and BioSample accession number SAMN24305868. The version described in this paper is version JAKDEZ010000000. The raw sequence reads were deposited in the Sequence Read Archive (SRA) under accession numbers SRR17327833 (Illumina) and SRR17322538 (PacBio).
